# Mastoid surface area-to-volume ratios in adult brazilian individuals

**DOI:** 10.5935/1808-8694.20130080

**Published:** 2015-10-08

**Authors:** Marco Antônio Rios Lima, Luciano Farage, Maria Cristina Lancia Cury, Fayez Bahmad Júnior

**Affiliations:** a MD, ENT (MSc. Student, Graduate Program on Health Sciences of the University of Brasília, Brasília, Brazil).; b PhD (MD, Radiologist, Advisor in the Graduate Program on Health Sciences of the University of Brasília, Brasília, Brazil).; c PhD (MD, ENT, Head of the Hyperbaric Medicine Service, Armed Forces Hospital, Brasília, Brazil).; d PhD (MD, ENT, Professor and Advisor in the Graduate Program on Health Sciences of the University of Brasília, Brasília, Brazil). University of Brasília.

**Keywords:** anatomy, mastoid, middle ear ventilation, tomography

## Abstract

Computed tomography-based measurements of mastoid surface area and volume have not been described for adult Brazilian individuals. These measurements add to the understanding of middle ear physiology, related diseases, and their impact on mastoid pneumatization.

**Objective:**

To check the ratio between mastoid surface area and volume.

**Method:**

This contemporary cross-sectional cohort study included 28 patients submitted to CT imaging of the mastoid. Measurements of surface area and volume were made based on serial CT scans and produced with the aid of software package Image Pro Plus.

**Results:**

Mastoid volumes ranged from 5.5 to 72.4 cm^3^. Surface areas ranged from 43.9 to 525.2 cm^2^. Surface areas varied linearly with volumes.

**Conclusion:**

Mastoid surface areas and volumes of adult Brazilian subjects followed a linear correlation, as also described in studies carried out in other countries. Mean surface areas and volumes were higher than previously published, unlike surface area-to-volume ratios, which were lower. Further studies with larger populations will provide evidence as to whether Brazilian subjects have larger surface areas and volumes than other populations.

## INTRODUCTION

The middle ear cleft is divided by the tympanic isthmus into anterior-inferior and posterior-superior compartments. The anterior-inferior compartment's main role is clearance, and it includes the protympanum, the mesotympanum, and the hypotympanum. The posterior-superior compartment comprehends the epitympanum and the retrotympanum, the aditus ad antrum and the antrum mastoideum, and the mastoid air cell system; it is primarily devoted to gas exchange. Both compartments are lined by the same respiratory mucosa. Gas exchanges are carried out through the air cell mucosa. Therefore, the total mucosal surface area has direct impact on gas exchange rates^1^.

The mastoid air cell system accounts for most of the volume of the middle ear cleft and, consequently, for most of the mucosa available for gas exchange^2^.

The role of the mastoid is yet to be clarified, despite the many theories on the matter. One of the main hypotheses states that the mastoid works as a gas reservoir used to compensate for pressure variations inside the middle ear together with the eustachian tube^2^, ^3^, ^4^, ^5^. According to Magnuson, the mastoid air cell system works as a thermal insulant to protect the middle ear against temperature fluctuations^5^. Others have described the mastoid as a no longer useful vestige of mammal evolution^6^.

Computed tomography (CT) is the best method to assess mastoid pneumatization^7^, ^8^. Various studies have looked into the volume of the adult mastoid, but few have considered the surface area of the mastoid. The latter is an important parameter in middle ear gas exchange through the mucosa^9^. The measurement of mastoid surface area and volume is relevant for the understanding of middle ear physiology and diseases, as it bears direct impact on mastoid pneumatization.

This study aimed to measure the surface area and volume of the mastoid using computed tomography scans of the temporal bones of adult Brazilians. Such measurements have not been described for Brazilian populations to date. Comparisons against data published in other countries are also included. Based on previous publications, the authors postulated that surface area and volume are linearly correlated^8^, ^10^.

## METHOD

This is a contemporary cross-sectional cohort study. Twenty-eight individuals without history of pediatric chronic otitis media or ear surgery were enrolled from a prospective study on the correlation between mastoid pneumatization and middle ear barotrauma in patients submitted to hyperbaric oxygen therapy.

All individuals gave informed consent and were informed of the risks inherent to their participation in the study. This study was approved by the Research Ethics Committee of the Brasília Armed Forces Hospital.

The subjects underwent bilateral pneumatic otoscopy and tympanometry to document healthy middle ears. Temporal bone CT scans were ordered for all patients.

Mastoid CT scans were taken with a 64-channel Lightspeed VCT (GE Healthcare, Milwaukee, WI, EUA) device, using a volume acquisition protocol with slices of 0.625 mm and slice intervals of 0.31 mm with a bone filter. Scans were produced in the axial view. Pixels measured 0.39 mm^2^.

The images were reprocessed on Osirix 64 with a reduced area of interest in slices of two millimeters, without excluding portions of the mastoid. All measurements were carried out in the same window, using a window level of 385 Hounsefield units (HU) and a window width of 3155 HU. Scans were saved on JPG format. Patient names were removed and the images were named after letters of the alphabet (A, B, C...) and categorized into right or left side scans.

A radiologist with 10 years of practical experience blinded for clinical data was called to assess the CT scans.

Surface and volume calculations were carried out using software package Image Pro Plus 6.0. The process used to analyze the scans included the following steps: tracing of the area of interest comprehended within the borders of the mastoid air cell system, including isolated cells and excluding the middle ear (the view showing the incudomalleolar joint was defined as the standard plane and the middle ear was considered to be located five planes below); stress poorly-defined borders of the air-bone gap when needed using tool Higauss (one use per slice); apply color mask on air cells to color them white and color bone or soft tissue black; calculate perimeter (cm) and area (cm^2^) of the region of interest for each slice; add the results from each slice per ear.

The area of each mastoid was calculated by multiplying the result of the summation of the perimeters of each slice by 0.2 cm (slice interval). The volume (cm^3^) of each mastoid was calculated by multiplying the result of the summation of the areas of each slice by 0.2 cm (slice interval). The analysis was carried out twice by the same researcher, who calculated the mean values found after two verifications of areas and volumes to use them as the final measurements. After the analysis was completed, the researcher was given the key to know the match between letters of the alphabet and patient data.

The procedure described above was also used in two previous studies to verify mastoid surface area and volume in adults^8^, ^10^. As in the study published by Park et al.^8^, only normal mastoids were included.

Parametric statistical tests were used due to the normal distribution pattern of the data. Linear regression was used to determine the correlations between (1) right and left mastoid volumes, (2) right and left mastoid surface areas, and (3) mastoid surface areas and volumes.

The database was built based on Excel®. Statistical analysis was carried out with software package SPSS 13^®^ (Statistical Package for the Social Sciences, Chicago, IL) for Windows®. Initially, the distribution of surface area and volume data points for each ear was analyzed. Outliers were identified based on interquartile range. According to this criterion, outliers reside outside the range defined by the following formula:

{Q1-[1.5x(Q3-Q1)]} ∪ {Q3+[1.5x(Q3-Q1)]}

Comparisons between mean values were carried out with *Student's t*-test for independent measurements (gender comparisons) or repeated measurements (right mastoid vs. left mastoid). Asymmetry analysis was based on the *t*-test for one sample, using zero as a test value (absence of asymmetry). Correlation tests (Pearson's) were carried out between variables measured through CT scans. The figures represent the values for each subject, the linear adjustment line, and the confidence intervals for the mean. Statistical significance was set at 5% (*p* < 0.05). All tests were two-tailed. Data was presented in the form of mean ± standard deviation (SD).

## RESULTS

Twenty-eight patients, 20 males (71.4%) and eight females (28.6%), with a mean age of 53 years (17 to 78 years) were enrolled in the study. No statistically significant differences were seen between the ages of male and female subjects (mean difference = 5.35 years; *p* = 0.471).

Four participants had two or more variables categorized as outliers. Tables 1 and 2 show the descriptive measurements of the variables analyzed in the study with the entire sample (n = 28) and with outliers excluded (n = 24). No difference was found between right and left mastoid surface areas or volumes.

**Table 1:** Variables assessed in the selected sample (n = 28).

Variable	Mean	SD	SE	IC 95%	
				Lower limit	Upper limit
Mastoid area					

RE	291.8	141.2	26.7	237.1	346.6
LE	303.6	133.6	25.3	251.7	355.4

Mastoid volume					

RE	31.9	17.9	3.4	25.0	38.8
LE	34.5	17.6	3.3	27.7	41.3

Area/volume ratio					

RE	9.5	1.9	0.4	8.8	10.3
LE	9.3	2.1	0.4	8.5	10.1

Area difference					

Absolute value	65.6	66.4	12.6	39.9	91.4
Right - Left	-11.7	93.5	17.7	-48.0	24.5
Variation (%)	-4.0	27.8	5.3	-14.8	6.8

Volume difference					

Absolute value	10.2	11.4	2.2	5.8	14.6
Right - Left	-2.6	15.2	2.9	-8.5	3.3
Variation (%)	-5.2	32.8	6.2	-18.0	7.5

SD: Standard deviation; SE: Standard error; CI: Confidence interval.

**Table 2:** Variables assessed in the sample (n = 24).

Variable	Mean	SD	SE	CI 95%	
				Lower limit	Upper limit
Mastoid area					

RE	304.9	143.4	29.3	244.4	365.5
LE	304.4	130.7	26.7	249.2	359.6

Mastoid volume					

RE	33.4	18.6	3.8	25.6	41.3
E	32.8	16.0	3.3	26.0	39.6

Area/volume ratio					

RE	9.6	1.9	0.4	8.8	10.4
LE	9.6	1.8	0.4	8.8	10.4

Area difference					

Absolute value	43.0	29.1	5.9	30.7	55.3
Right - Left	0.5	52.7	10.8	-21.7	22.8
Variation (%)	-0.9	17.8	3.6	-8.5	6.6

Volume difference					

Absolute value	6.3	5.3	1.1	4.0	8.5
Right - Left	0.6	8.3	1.7	-2.9	4.1
Variation (%)	-1.0	21.8	4.5	-10.2	8.3

SD: Standard deviation; SE: Standard error; CI: Confidence interval.

When the entire sample was considered (n = 28), the right and left mastoid volumes were 31.9 ± 17.9 cm^3^ (5.5 to 72.4) and 34.5 ± 17.6 cm^3^ (6.2 to 68) respectively (*p* = 0.4; *Student's t-test* for paired samples). Right and left mastoid surface areas were 291.8 ± 141.2 cm^2^ (43.9 to 525.2) and 303.6 ± 133.6 cm^2^ (45 to 520.4) respectively (*p* = 0.56). The surface area-to-volume ratios for the right and left mastoids were 9.5 ± 1.9 cm-^1^ (6.8 to 13.7) and 9.3 ± 2.1 cm^-1^ (5 to 13.8) respectively (*p* = 0.32). The correlation ratios for surface area and volume for the left and right mastoids were 0.88 and 0.92 respectively, yielding a global ratio (all mastoids considered) of 0.9 (Table 1).

In the sample net of outliers (n = 24), the right and left mastoid volumes were 33.4 ± 18.6 cm^3^ (5.5-72.4) and 32.8 ± 16 cm^3^ (6.1 to 66.9) respectively (*p* = 0.71; *Student's t*-test for paired samples). Right and left mastoid surface areas were 304.9 ± 143.4 cm^2^ (43.9 to 525.2) and 304.4 ± 130.7 cm^2^ (44.9 to 469.8) respectively (*p* = 0.96). The surface area-to-volume ratios for the right and left mastoids were 9.6 ± 1.9 cm^-1^ (6.8 to 13.7) and 9.6 ± 1.8 cm^-1^ (6.9 to 13.8) respectively (*p* = 0.91). The correlation ratios for surface area and volume for the left and right mastoids were 0.9 and 0.92 respectively, yielding a global ratio (all mastoids considered) of 0.91 (Table 2).

**Figure 1:**
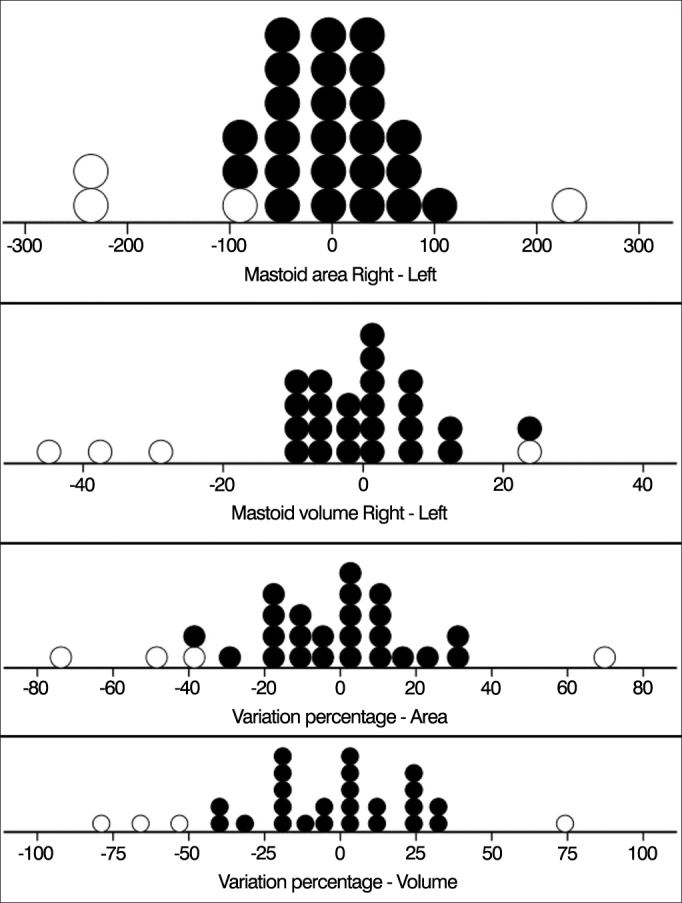
Distribution of asymmetry values measured in the sample. Outliers are represented by open circles (n = 28).

In order to better describe asymmetry measurements (differences of surface area and volume), Figure 1 shows individual values. Negative values mean greater values for left ears and positive values mean greater values for right ears. The mean difference between surface area or volume values for right and left mastoids was not significantly different from 0 (*p* > 0.376 in all cases). This analysis was carried out for the entire sample (n = 28) and for the sample with outliers excluded (n = 24).

**Figure 2:**
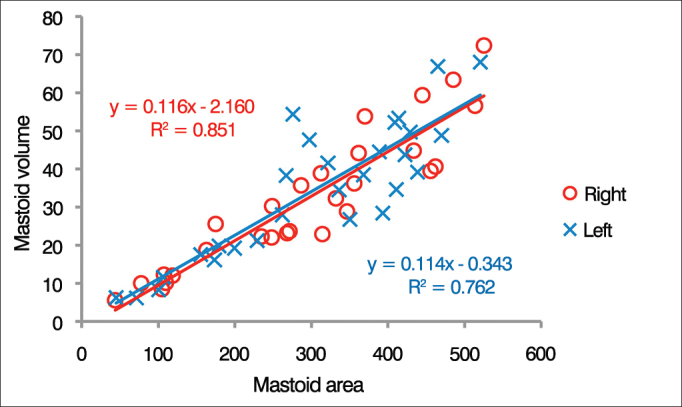
Distribution of individual surface area-to-volume ratios. The lines represent the linear regression of the data for each ear as defined by the formula presented in the graph (n = 28).

Figure 2 shows the surface areas of right and left mastoids as a function of their respective volumes (n = 28). A linear correlation was found between mastoid surface area and volume. For left and right ears, 76% and 85% of the variance in mastoid surface area was explained by the volume regressions respective of each ear.

**Figure 3:**
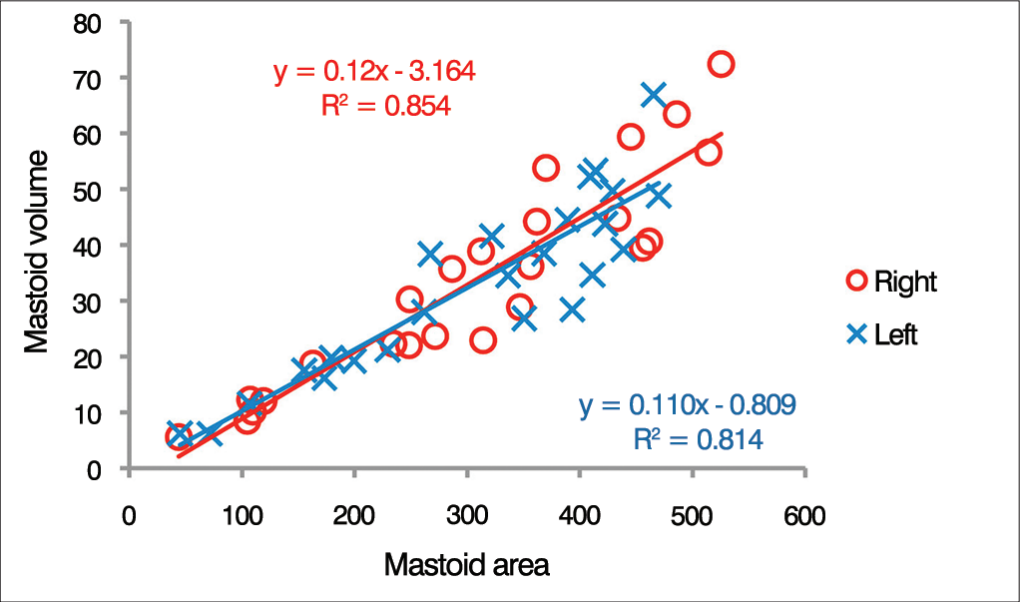
Distribution of individual surface area-to-volume ratios. The lines represent the linear regression of the data for each ear as defined by the formula presented in the graph (n = 24).

Figure 3 shows the surface areas of right and left mastoids as a function of their respective volumes (n = 24). A linear correlation was found between mastoid surface area and volume. For left and right ears, 81% and 85 % of the variance in mastoid surface area was explained by the volume regressions respective of each ear.

**Figure 4:**
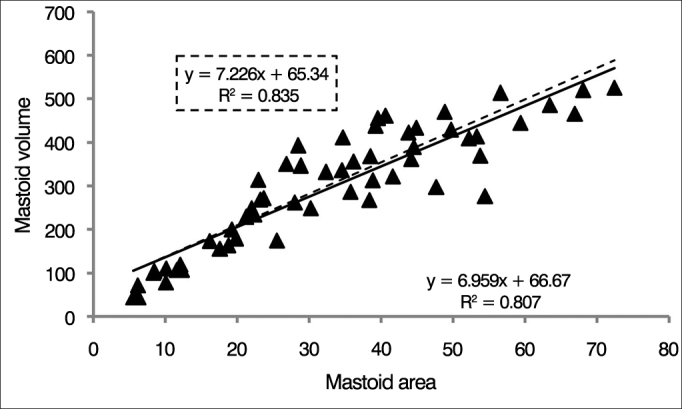
Distribution of individual surface area-to-volume ratios. The lines represent the linear regression of the data for each ear as defined by the formula presented in the graph. The traced line and the formula highlighted by the traced line represent the values calculated for the sample without outliers (n = 28).

Figure 4 shows the surface areas of all mastoids as a function of their volumes. A linear correlation was found between mastoid surface area and volume. For all ears (n = 28), 80% of the variance in mastoid surface area was explained by volume regressions. In the sample net of outliers (n = 24), the value was 83%.

## DISCUSSION

The eustachian tube, the tympanic cavity, and the mastoid are part of a complex system in which various mechanisms are combined to ensure proper middle ear ventilation^9^. Measurements of surface area and volume are of great importance in the study of mastoid physiology and pathogenesis. The area used for gas exchange is directly related to the mucosal surface area. Nonetheless, mucosal surface may be underestimated, as CT scans cannot detect the numerous folds present in the mucosa of the mastoid^5^. Therefore, normal and altered (ossified or opacified) mastoids may have different gas exchange capabilities despite equal measurements of surface area and volume as inferred from CT scans.

Many authors have described correlations between poor mastoid pneumatization and diseases of the middle ear^11^, ^12^. However, there is no consensus as to whether people with smaller mastoid volumes would be at greater risk of having middle ear disease, or if middle ear diseases could hamper the development of the mastoid air cell system^13^.

The main discussion regarding the role of the mastoid is centered on whether the mastoid works as a gas reservoir. Most authors believe the mastoid works as a gas reservoir, acting passively to counteract pressure fluctuations in the middle ear together with the eustachian tube^1^, ^2^, ^3^, ^4^, ^14^. Others have refuted this idea^6^, ^15^, ^16^. Some authors believe the mastoid has an active two-way role in gas and fluid exchange^5^, ^17^, despite the lack of significant experimental evidence^2^. Many experimental models have been tried, limited by blood perfusion and gas diffusion, but their results have been contradictory^14^, ^15^, ^16^.

The degree of mastoid pneumatization has been assessed through various methods: immersion in fluid, Boyle's law, acoustics, and imaging^18^, ^19^. Due to the difficulty accessing mastoid pneumatization directly, imaging has been the most frequently used method. Temporal bone CT scans provide more accurate assessments of mastoid volumes than temporal bone X-rays^7^, ^8^.

Coulhon et al.^20^ found a good correlation between mastoid pneumatization estimated by X-rays using the planimetric approach and mastoid volume calculated from CT scans (r = 0.95). Conversely, Todd et al.^7^ found more modest correlation ratios between X-rays and CT scans (r = 0.57-0.74). Temporal bone X-rays are reliable when patients of similar ages are compared, but they cannot be used to assess actual or air mastoid volumes or surface areas^9^.

Only a few Brazilian authors have quantitatively looked into mastoid pneumatization^21^, ^22^. Albernaz^21^ analyzed 100 pairs of temporal bones of mostly Brazilian individuals and assessed the value of the occipital position in the radiographic study of the mastoid. The author compared right and left mastoids and found identical pneumatization on both sides in 82 % of the pairs, relative agreement between both sides in 17%, and absolute disagreement in 1% of the cases. However, the author did not report absolute values of mastoid surface area. Bento et al.^22^ correlated mastoid pneumatization on X-rays to graft take in 80 patients with chronic otitis media submitted to type I tympanoplasty. The authors reported a mean mastoid surface area of 7.62 cm^2^ (1.82 to 27.4). We believe this is the first Brazilian study to report measurements of mastoid surface area and volume verified with CT.

Strong correlations were found between the right and left mastoid volumes (r = 0.65), surface areas (r = 0.79), and surface area-to-volume ratios (r = 0.78) of the subjects included (n = 28) in this study (Table 3). The sample net of outliers (n = 24) revealed even stronger correlation coefficients: (r = 0.89), (r = 0.93), and (r = 0.83) respectively (Table 4). The low number of individuals included in this study (n = 28) makes it harder to tell whether the four patients with discrepant findings were true outliers or if they accounted for the normal variation within the studied population.

**Table 3:** CT parameters correlation assessment (n = 28).

	Area LE	Volume RE	Volume LE	A/V RE	A/V LE
Area RE					

r	0.792	0.926	0.599	0.016	0.256
*p*-value	< 0.001	< 0.001	0.001	0.935	0.181

Area LE					

r	-	0.722	0.885	0.051	0.032
*p*-value	-	< 0.001	< 0.001	0.794	0.870

Volume RE					

r	-	-	0.656	-0.322	-0.020
*p*-value	-	-	< 0.001	0.088	0.917

Volume LE					

r	-	-	-	-0.262	-0.387
*p*-value	-	-	-	0.169	0.038

A/V RE					

r	-	-	-	-	0.785
*p*-value	-	-	-	-	< 0.001

**Table 4:** CT parameters correlation assessment (n = 24).

	Area LE	Volume RE	Volume LE	A/V RE	A/V LE
Area RE					

r	0.930	0.924	0.855	-0.170	0.020
*p*-value	< 0.001	< 0.001	< 0.001	0.427	0.927

Area RE					

r	-	0.839	0.903	-0.102	0.034
*p*-value	-	< 0.001	< 0.001	0.635	0.875

Volume RE					

r	-	-	0.896	-0.496	-0.270
*p*-value	-	-	< 0.001	0.014	0.202

Volume LE					

r	-	-	-	-0.411	-0.365
*p*-value	-	-	-	0.046	0.079

A/V RE					

r	-	-	-	-	0.833
*p*-value	-	-	-	-	< 0.001

Swarts et al.^10^ reported correlation coefficients similar to the values found for the sample without outliers (n = 24) for right and left mastoid surface area and volume (r = 0.87 for surface area and volume). Considering the entire sample (n = 28), there was more asymmetry in the measurements of surface area and volume of right and left mastoids, particularly in volume (r = 0.65). Yet, symmetry prevailed. For bilateral structures, strong correlations between the measurements of each side (bilateral symmetry) indicate significant genetic contribution, whereas weak correlations between these measurements (bilateral asymmetry) are suggestive of predominantly environmental contribution to the assessed structure^23^. This fact reports back to two theories on mastoid development^24^, ^25^. The theory of inheritance described by Diamant^24^ states that the degree of mastoid pneumatization is genetically determined, consequently yielding higher levels of symmetry. According to Wittmaack^25^, the degree of mastoid pneumatization is influenced by episodes of otitis media or tubal disorders during childhood, which could explain asymmetries. As the population included in this study was made up of adults with healthy ears without history of pediatric chronic otitis media, the reported degree of volume symmetry could be explained by the genetic theory described by Diamant^24^. However, reports of chronic otitis media occurred during childhood could be subject to memory bias and confound the analysis^10^. Other factors could affect genetically programmed expression and lead to various degrees of asymmetry^13^.

In the complete sample (n = 28) and in the sample without outliers (n = 24), the mean values found for surface area (297.7 cm^2^ and 304.6 cm^2^ respectively) and volume (33.2 cm^3^ and 33.1 cm^3^ respectively) for all ears were greater than the values reported in previous studies^8^, ^10^. Conversely, the mean surface area-to-volume ratio was lower than previously reported for the complete sample (n = 28) and for the sample without outliers (n = 24) (9.4 cm^-1^ and 9.6 cm^-1^ respectively)^8^, ^10^. Magnuson^5^ claimed gas exchange capacity was directly correlated with the surface area-to-volume ratio. However, recent studies have played down the importance of the surface area-to-volume ratio in gas exchange, as ratios appear to be somewhat constant^13^, ^16^. Csakanyi et al.^9^ compared measurements of mastoid area and volume in children with and without otitis media with effusion (OME) and contradictorily reported greater surface area-to-volume ratios in children with OME. Therefore, more studies are required to clarify the actual meaning and significance of the surface area-to-volume ratio in mastoid physiology and pathogenesis.

Some specific factors may impact the extent of mastoid pneumatization. Reduced pneumatization of the temporal bone is evident in craniofacial disorders such as achondroplasia, Pierre Robin syndrome, and Crouzon syndrome. Cystic fibrosis results in increased temporal bone pneumatization^26^. In this study, none of the patients had specific syndromes or diseases to explain for the high mean surface areas and volumes reported.

## CONCLUSION

The mastoid surface areas and volumes measured in adult Brazilians follow a linear correlation, as described in studies carried out in other countries. More studies with larger samples may provide clarification on whether the mean mastoid surface area and mean mastoid volume of the Brazilian population is higher than the mean values found for other populations.
